# Increased Education is Associated with Decreased Compliance in an Urban Multi-Ethnic Lupus Cohort

**DOI:** 10.4172/2155-9899.1000215

**Published:** 2014-05-26

**Authors:** Rachel Gross, Jennifer Graybill, Dawn Wahezi, Nicole C. Jordan, Chaim Putterman, Irene Blanco

**Affiliations:** 1 Division of Rheumatology, Albert Einstein College of Medicine, Bronx, NY, USA; 2 Division of Pediatric Rheumatology, Albert Einstein College of Medicine, Bronx, NY, USA

**Keywords:** Systemic lupus erythematosus, Medication compliance, Racial and ethnic minorities

## Abstract

**Objectives:**

To investigate the factors associated with medication compliance in a multi-ethnic population of patients with systemic lupus erythematosus in an urban community.

**Methods:**

We surveyed patients in our cohort using the standardized measures of the Compliance-Questionnaire-Rheumatology (CQR), the Beliefs about Medications Questionnaire (BMQ), as well as patient self-reported compliance. Demographic and clinical characteristics of compliant and non-compliant patients underwent bivariate analysis. A multivariate analysis was then performed on variables of interest.

**Results:**

Of the 94 patients who agreed to participate in the survey, 89 fully completed each questionnaire. Overall, 48% of patients were compliant by CQR. In multivariate analyses, higher education level was associated with non-compliance. Spanish-speaking patients and those with an income of greater than $15,000 per year were more likely to be compliant.

**Conclusions:**

In this urban lupus population, several factors may influence medication compliance. Factors associated with non-compliance are not what have been found in other populations. Further studies looking into specific reasons for certain areas of non-compliance as well as addressing these issues will be important in both treatment and outcomes in lupus patients in implementing appropriate interventions.

## Background

Systemic Lupus Erythematosus (SLE) is a chronic autoimmune disease that affects multiple organ systems. In the US, SLE disproportionately affects non-Caucasians, particularly African Americans and Hispanics. Not only is the prevalence of lupus higher in minority communities, these patients also tend to have a greater burden of SLE activity and organ damage [1–5]. Adequate treatment is essential to provide good outcomes.

While genetic predisposition, environment, and socioeconomic status influence outcomes in SLE, it is likely that other factors affect disease activity as well [2,3,6]. Medication non-compliance may help to explain differences in lupus activity that are not otherwise explained. Medication compliance rates for patients with SLE range widely from 54–93% [7,8]. While traditional risk factors such as race are associated with renal disease, this association may be mediated by compliance to treatment regimens [9]. Some authors though have found that poor compliance is associated with both race and outcomes [10].

Few have investigated the specific barriers to medication compliance in SLE. A qualitative study in the UK found that: patients felt that SLE should be controlled using alternative methods; had a fear of side effects; and noted poor patient-physician communication [11]. In a US study of SLE and rheumatoid arthritis patients, patients who had difficulty with compliance reported: a fear of side effects; a lack of efficacy, financial costs of medications, and difficulty navigating the healthcare system [12].

Even though these studies included patients of different racial and ethnic origins, they were too small to investigate differences in compliance by these parameters. Mosley-Williams et al. explored potential compliance barriers between a group of African American and Caucasian women with lupus. While there was no difference between the groups with regards to medication compliance, African American participants were more likely to have concerns regarding their medications [13].

Although there were no racial differences in the above study, compliance is still an issue in the SLE population and has not been well-studied in minority groups specifically. Therefore, we sought to elucidate potential barriers to treatment in an African American/Afro-Caribbean and Hispanic SLE population. We performed a pilot cross-sectional survey of medication compliance using the validated Compliance-Questionnaire-Rheumatology (CQR) where we examined multiple variables that may contribute to medication non-compliance in our minority lupus population.

## Patients and Methods

### Population

Patients were recruited from the Einstein Lupus Cohort (ELC), a prospective multi-ethnic cohort followed at Albert Einstein College of Medicine, Bronx, NY. ELC patients are predominantly African American, Afro-Caribbean and Hispanic. Our Afro-Caribbean and Hispanic patients mostly hail from Jamaica, the Dominican Republic and Puerto Rico. However there are many patients from other countries throughout the Caribbean as well as Latin America. While our patients may identify with these places, approximately 80% of the cohort was born in the US and US territories.

Approval was obtained from the Committee on Clinical Investigations, the designated Institutional Review Board and all patients provided consent for participation in accordance to the Declaration of Helsinki. Study recruitment took place between August 2010 and April 2011. Participants in the study were ≥ 18 years and met at least 4 American College of Rheumatology (ACR) criteria for the diagnosis of SLE. Participants were to have adequate language proficiency in either English or Spanish.

### Patient recruitment and questionnaire

All members of the cohort seen between the above dates were invited to participate by a study investigator. Once consent was obtained, the participants independently completed a comprehensive written questionnaire. Questionnaires were completed anonymously in the patient’s preferred language, either English or Spanish. The questionnaire included the following components:

1. *Compliance-Questionnaire-Rheumatology (CQR)* - a 19 item questionnaire validated in patients with rheumatic diseases. Patients rate items based on a 4-point Likert scale, with responses ranging from 1 (do not agree at all) to 4 (agree very much). The sum of the weighted responses is then compared to a predetermined cutoff score of −0.585, where those with this score or less are at least 80% compliant. This has been found to have a sensitivity and specificity of 95% and 62% respectively to detect medication noncompliance [14]. We used the CQR as the main determinant of compliance in our study. All patients’ CQR scores were calculated and dichotomized to “Non-Compliant” or “Compliant” based on the cut-off. Since incomplete CQR questionnaires are not able to be scored, patients with missing responses were excluded from the analysis.
2. *Self-reported compliance* - Patients were asked how often they forgot or chose not to take a prescribed dose of a medication. Responses included: never/rarely (1 or two times a month), sometimes (once a week), often (more than once a week). Patients who answered that they missed their medication once a week or more were classified as non-compliant by this self-report measure.
3. *Beliefs About Medicines Questionnaire (BMQ)* – a 10 item validated questionnaire that measures patient’s beliefs about the necessity of (5 items) and concerns regarding (5 items) prescribed medications [15]. Patients are asked to rate their agreement with statements on a 5 point Likert scale: 1 (strongly agree) to 5 (strongly disagree). The total scores for necessity and concern scales range from 5–25, with higher scores indicating stronger beliefs of the necessity of a medication or stronger concerns about a medication.
4. Patients were asked to elaborate on the reasons they missed doses of their medications. Patients were given multiple response questions and asked to “circle all those that apply.” They were given the following choices: 1. Forgot to take medication; 2. Afraid of side effects or thought the medication was toxic/harmful; 3. Felt too sick or ill; 4. Felt medication was not helping you; 5. Felt depressed or overwhelmed, 6. Felt good/did not feel it was important to take the medication; 7. Ran out of medication; 8. Other.
5. Demographic information collected included: age, gender, race, ethnicity household income, level of education, country of origin, primary language (English or Spanish) and disease duration. Additionally, disease activity was measured during the visit using the SLE Disease Activity Index (SLEDAI) and medication information was collected by the rheumatologist evaluating the patient, as is standard protocol at ELC visits.

### Statistical analysis

All statistical analyses were performed using STATA 10.1 (College Station, TX). Demographic and clinical characteristics of compliant and non-compliant participants underwent bivariate analyses using the Chi-square test for categorical variables and the Mann-Whitney U test for not-normally distributed continuous variables. Variables of interest from the bivariate analysis and those with a p-value <0.20 were further evaluated using a multivariate logistic regression model to see which were associated with non-compliance in our cohort. All variables were tested alone and in groups and all models were tested for the assumptions of logistic regression modeling and collinearity.

## Results

One hundred and eight ELC patients were seen between August 1, 2010 and April 30, 2010. Ninety-four of 108 patients agreed to participate (87% participation rate). Five patients did not have complete CQRs on their surveys and were excluded, leaving 89 patients with completed questionnaires that were included in our analysis.

The median age of the study population was 37 years (IQR: 23). Participants were predominantly female (92%), and the majority were Hispanic (47%), African American (29%) or Afro-Caribbean (19%). Fifty-one percent of patients reported an education level beyond a high school diploma. Most had a yearly household income of less than $15,000 per year. Additional clinical and demographic characteristics of study participants are summarized in Table 1.

Overall, only 48% of patients (43/89) were compliant as measured by the CQR. However, when patients were asked to self report their compliance with medications, this rate increased to 68%, where 60 of the 89 patients reported missing medication “never” or “rarely” (i.e. less than one time/week).

The bivariate analyses of the individual variables associated with compliance and non-compliance are shown in Table 2. We found that non-compliant patients had significantly higher BMQ concerns scores, meaning that they had more concerns about their medications (p=0.02). While the BMQ score was the only variable on bivariate studies to show statistical significance, education trended towards significance. 59.5% of patients with a high school education or above were non compliant with medications compared to 39% of those with less education (p=0.06).

Several variables had a p-value below the a priori threshold of 0.20 for inclusion in the multivariate analysis. Solely Spanish speaking (OR: 2.78; 95% CI: 0.67, 11.57), patients taking anti-malarial medication (OR: 1.91; 95% CI: 0.71, 5.16), and surprisingly, patients reporting more than one medication side effect per week (OR: 1.84; 95% CI: 0.76, 5.16) were potentially more likely to be compliant. In contrast, those with a yearly income less than $15,000 (OR: 0.48; 95% CI: 0.19, 1.25) and those taking mycophenolate mofetil (OR: 0.31; 95% CI: 0.08, 1.23) were less likely to be complaint. Several factors were not associated with compliance or did not have p-values below threshold for the multivariate analysis. Further details are shown in Table 2.

We constructed a multivariate logistic regression model using variables of interest from the bivariate analysis including BMQ medication concerns, and the variables listed above. We also included potential confounders: age, race, gender and insurance status as well as SLEDAI score and disease duration regardless of the p-value in the bivariate analyses. Interestingly, when adjusted for the above variables, patients with more than a high school diploma were 78% less likely to be compliant (p=0.014). Another surprising finding was that Spanish speakers (p=0.04) were more likely to be compliant. Additionally, those with income of greater than $15,000 per year were more likely to be compliant (p=0.045). Those with more medication concerns (BMQ concerns score) trended towards predicting decreased compliance (p=0.058) (Table 3).

In order to further investigate this unanticipated negative association between compliance and education, we looked at other variables that may be associated specifically with education in this population. As to be expected, patients with an education past high school had higher incomes than those with less years of education (p=0.044). Interestingly, those with at least a high school education and above had higher median BMQ scores with regards to concerns about their medications that trended towards significance (14.0 v 11.5, p=0.07) while there was no difference in the patients BMQ necessity scores (11.0 v 10.0, p=0.91). There was no difference in median SLEDAI scores (2 v 2, p=0.85), and patients with higher levels of education were more likely to have Medicare coverage or private insurance (p=0.050).

We next sought to investigate self-reported reasons for non-compliance. Although 68% percent reported that they “Never” or “Rarely” missed medications, 43 of the 81 patients that responded to the question did admit to forgetting doses. Twenty-seven patients also admit to missing doses when they run out of their medications. Interestingly, 16 of 81 stated that they missed doses when they “felt good/did not feel it was important to take medication.” Further results are presented in Table 4. When we studied these responses and how they relate to education in this population, those with more than a high school education where more likely to report missing doses because they forgot to take the medication than those with less years of education (67.5% v 32.5% p=0.002). None of the other responses were significantly associated with education.

## Discussion

Using a validated medication compliance questionnaire, we found that surprisingly just over half of our patients (52%) in this minority lupus cohort were compliant with their medications. Higher levels of education were associated with decreased compliance, as were the concerns for possible side effects. Those that did not speak English and had higher incomes were more likely to be compliant. When looking into the self-reported reasons for non-compliance, more than half of our patients admitted to forgetting doses of medication (53%).

The degree of medication compliance is notably lower than what has been found in other populations with rheumatic diseases. Van den Bemt et al. in a study using the CQR in patients with rheumatoid arthritis, found a 68% compliance rate with regards to Disease Modifying Anti-Rheumatic Drugs usage [16]. In this Dutch study non-compliance was weakly associated with disease duration, and the perceived side effects and necessity of the medication. The reasons for non-compliance in our group, however, appear to be somewhat different.

An interesting and counter-intuitive finding in our study was that patients with higher levels of education were 78% less likely to be compliant with medication. These lower levels of compliance among the more highly educated may be due to a combination of factors. It may be that patients with more education are more inclined to question the doctor’s decisions with medication choices (e.g. Is the doctor telling me all the risks of the medication or giving me all the possible options for treatment?). Consistent with this hypothesis is the study by Nived et al., who previously reported that lupus patients with a higher level of education were more likely to self-adjust the dose of their glucocorticoid prescription [17]. Lupus patients with higher education may feel they know their disease and treatment options, and are capable of making an educated decision about what is best.

Patients with more education might be more likely to research both their disease and medications in greater detail. With the increasing availability of the internet, patients now have significant access to information about their disease and treatment options. Kowalczyk and Draper found that over the last 10 years, cancer patients have become increasingly dependent on outside sources for medical information, and are now less dependent on their physician [18]. Recent estimates show that over 60% of patients attending rheumatology clinics research their symptoms and diseases on-line prior to seeing a rheumatologist [19]. Web searching may make patients feel more empowered and this empowerment may lead to independent treatment decisions without the consultation of a physician [20]. If this is the case, what may actually worsen the situation is that physicians rarely, if ever, help steer patients to reputable websites [21]. Therefore, patients are likely encountering enormous amounts of information without the knowledge to decipher what information is in fact correct and could consequently be making poor decisions based on erroneous information [22].

While this is an interesting possibility, we did not address electronic information sources as a potential mediator of compliance in our study. For the most part patients stated that the missed doses were simply due to forgetting medication. However, the problem may be more extensive especially given that those patients with higher education have (though not statistically significant) higher BMQ side effect concerns score. It is possible that they therefore know more and are more concerned about their medications.

It should be noted that although the more educated patients in our cohort were less likely to be compliant, these patients were still of low socioeconomic status (SES). Low income itself was also independently associated with medication non-compliance. Alarmingly, nearly two thirds of our patients reported having a yearly household income of less than $15,000 per year. In fact, the most recent U.S. Census reports that almost 30% of the population in the Bronx lives below the poverty line [23]. Poverty and low SES have been linked to poor outcomes and increased mortality in lupus patients, independent of race and ethnicity [24–26]. In this cohort with such limited monetary support, it may be that patients have to choose between taking their medication and being able to provide basic necessities such as food and shelter. Understandably the precise interplay between income and compliance will likely vary for the individual patient. Nevertheless it is important for the physician to discuss income as a possible barrier to compliance given there are potential solutions such as industry and hospital based assistance programs.

In our study, patients with Spanish as their primary language were more likely to be compliant. This is in contrast to previous research which cites language barriers as a reason for decreased compliance and dissatisfaction with health care systems in patients with rheumatic diseases [12]. However, other investigators have reported similar results. In a group of minority schizophrenic patients, Hispanics with limited English proficiency were actually more compliant than Hispanics that spoke English well [27]. A study conducted at another New York medical school, with a similar Hispanic population, found that while limited English was associated with decreased overall satisfaction with care, it was not associated with decreased compliance [28]. This may be due to cultural differences between non-acculturated and acculturated/US-born Hispanics. It is possible that our non-acculturated, Spanish speaking patients are less likely to question the advice of their physician where they may prefer a somewhat more paternalistic relationship with their physicians as opposed to other US ethnic groups [29].

While there was no difference between the groups with regards to the necessity of their medications, the non-complaint group had significantly higher BMQ concerns score on bivariate analyses that trended toward significance on the multivariate analysis. It stands to reason that the group with the highest levels of concern about medications and their possible side effects would more likely be noncompliant. However this was not the case in van den Bemt’s study where concerns about medication, as measured by the BMQ in RA patients, were not associated with compliance [16].

When we looked into the reasons patients provided for noncompliance, the primary response was not remembering their medication. Interestingly, those with more than a high school education were more likely to report they forgot to take their medication. Our study did not pursue reasons for forgetting medications, but we feel there are several potential explanations. Patients with more education may have more opportunities for employment and therefore a more complex schedule that leads to missing medications. Nevertheless, even with higher levels of education our patients still have limited income. This possibly causes our patients to spend more time concentrated on supporting themselves and less time on their chronic diseases.

Our study was a pilot study to determine which potential variables influence compliance in our predominantly minority, urban SLE population. While we had a very good response rate of 87%, this may have introduced bias into our sample. It is possible that this high rate stems from surveying patients already participating in our SLE cohort. Because they already participate in the cohort, it is possible that they are more complaint than the average clinic patient. This is a troublesome possibility given that we have such a high noncompliance rate in our own cohort.

The relatively small sample size limits how much we can extrapolate from this data. We see in our multivariate models that the confidence intervals for our significant variables are wide and therefore imprecise. In the future we will need to expand our study to a larger population to better estimate the association between compliance, education, income and language preference. However the results from this pilot are intriguing and warrant further investigation.

Although compliance was determined by a validated questionnaire and not direct self-report measures, reporting bias may still be present, especially since the survey was administered in a clinical setting. Also, though the CQR has been validated in patients with chronic rheumatic diseases such as rheumatoid arthritis, it has not been specifically validated in patients with SLE and therefore it may not truly capture medication compliance in this population. However, de Klerk et al. did study patients with rheumatic diseases who face similar situations as lupus patients such as having chronic medical conditions, long term medication use including steroid use, and medications with potentially harmful side effects [14]. Regardless, despite our limitations we have found very interesting and sometimes counterintuitive associations between certain variables and compliance in this minority population.

In summary, we found that medication compliance was influenced by several factors in our multi-ethnic cohort of lupus patients. More research is needed to better understand reasons for non-compliance such as patients’ subjective choices and to identify specific interventions to improve compliance in this population. We also need to further explore how patients understand their disease and how they obtain information to make decisions outside of what is provided through patient-physician interaction. This knowledge will allow physicians and other members of the health care team to better understand and communicate with our patients, improve patient compliance, and hopefully contribute to better patient outcomes.

## Figures and Tables

**Table 1:** Demographic and clinical characteristics of the participants (N = 89).

**Median age, yrs (IQR)**	37 (23)

**Female Gender (%)**	82 (92)

**Race (%)**	

Hispanic	42 (47)
African American	26 (29)
Afro-Caribbean	17 (19)
Asian	3 (3)
Other	1 (1)

**English Speakers (%)**	79 (89)

**Born in the US or US territory (%)**	63 (71)

**Median disease duration, yrs (IQR)**	8 (12)

**Income (%)**	

< 15,000/yr	44 (63)
15–35,000/yr	19 (27)
>35,000/yr	7 (10)

**Education (%)**	

Below high school	23 (28)
Graduate high school/GED	18 (22)
Education beyond high school	42 (51)

**Hospital (%)**	

Clinic at private hospital	66 (74)
Clinic at public hospital	23 (26)

**SLEDAI, median (IQR)**	2 (3)

**Disease duration, median (IQR)**	8 (12)

**Non-compliance as reflected by a CQR <80 (%)**	46 (52)

**Table 2:** Bivariate analysis of individual variables associated with compliance and non-compliance.

	Noncompliant N=46	Compliant N=43	p-value
Median age, yrs (IQR)	36 (27)	38 (21)	0.88
Female (%)	52.4	47.6	0.70
African American or Afro-Caribbean (%)	55.8	44.2	0.82
Solely Spanish speaking (%)	30.0	70.0	0.18*
Education above High School (%)	59.5	40.5	0.06*
Income <$15,000 (%)	54.5	45.5	0.13*
Medicare/Private Insurance (%)	57.1	42.9	0.49
Disease Duration, yrs (IQR)	9 (12)	7 (11)	0.22
SLEDAI, points (IQR)	2 (2)	3 (5)	0.37
Mycophenolate mofetil use (%)	75.0	25.0	0.12*
Other DMARD use (%)	57.1	42.9	0.51
Hydroxychloroquine use (%)	47.8	52.2	0.19*
Self reported as compliant (miss medications < 1x/week) (%)	51.7	48.3	0.87
Mean BMQ concerns score (±SD)	14.5 (0.62)	12.3 (0.60)	0.02*
Median BMQ necessity score (±SD)	10.8 (3.9)	11.0 (4.1)	0.82
Side effects ≥ 1x/wk (%)	44.1	55.9	0.18*

* These variables where included in the multivariate analysis to determine which of the above were most associated with compliance when adjusting for other variables.

**Table 3:** Results of multivariate analyses using logistic regression models to determine the association of specific variables to compliance.

	Odds ratio	P-value	95% Confidence Interval
Education >high school	0.224	0.014	0.068 – 0.742
Language (Spanish)	12.445	0.040	1.124 – 137.729
Income >$15,000	3.478	0.045	1.027 – 11.782
Concerns for side effects	0.864	0.058	0.742 – 1.005

**Table 4:** Responses to why medication doses were missed. N=81*

Possible choices	# of responses
Forgot to take medication	43
Afraid of side effects or thought medication was toxic/harmful	7
Felt too sick or ill	10
Felt medication was not helping you	4
Felt depressed or overwhelmed	15
Felt good/did not feel it was important to take the medication	16
Ran out of medication	27

* Patients were allowed to pick more than one response

## Key Messages

- Patients with a higher level of education were less likely to be compliant.
- Spanish speakers and patients with an income of greater than $15,000 per year were more likely to be complaint.
- Patients with concerns about their medication trended towards being non-compliant
